# Evaluating The Impact of Risk Factors on Birth Weight and Gestational Age: A Multilevel Joint Modeling Approach 

**DOI:** 10.22074/ijfs.2018.5330

**Published:** 2018-03-18

**Authors:** Payam Amini, Abbas Moghimbeigi, Farid Zayeri, Hossein Mahjub, Saman Maroufizadeh, Reza-Omani Samani

**Affiliations:** 1 Department of Biostatistics and Epidemiology, School of Public Health, Hamadan University of Medical Sciences, Hamadan, Iran; 2 Modeling of Noncomunicable Disease Research Center, Department of Biostatistics, Faculty of Public Health, Hamadan University of Medical Sciences, Hamadan, Iran; 3 Department of Biostatistics, Proteomics Research Center, School of Paramedical Sciences, Shahid Beheshti University of Medical Sciences, Tehran, Iran; 4 Research Center for Health Sciences, Department of Biostatistics, Faculty of Public Health, Hamadan University of Medical Sciences, Hamadan, Iran; 5 Department of Epidemiology and Reproductive Health, Reproductive Epidemiology Research Center, Royan Institute for Reproductive Biomedicine, ACECR, Tehran, Iran

**Keywords:** Birth Weight, Gestational Age, Multilevel, Statistical Model

## Abstract

**Background:**

Abnormalities in birth weight and gestational age cause several adverse maternal and infant out- comes. Our study aims to determine the potential factors that affect birth weight and gestational age, and their association.

**Materials and Methods:**

We conducted this cross-sectional study of 4415 pregnant women in Tehran, Iran, from July 6-21, 2015. Joint multilevel multiple logistic regression was used in the analysis with demographic and obstetrical variables at the first level, and the hospitals at the second level.

**Results:**

We observed the following prevalence rates: preterm (5.5%), term (94%), and postterm (0.5%). Low birth weight (LBW) had a prevalence rate of 4.8%, whereas the prevalence rate for normal weight was 92.4, and 2.8% for macrosomia. Compared to term, older mother’s age [odds ratio (OR)=1.04, 95% confidence interval (CI): 1.02-1.07], preeclampsia (OR=4.14, 95% CI: 2.71-6.31), multiple pregnancy (OR=18.04, 95% CI: 9.75- 33.38), and use of assisted reproductive technology (ART) (OR=2.47, 95% CI: 1.64-33.73) were associated with preterm birth. Better socioeconomic status (SES) was responsible for decreased odds for postterm birth com- pared to term birth (OR=0.53, 95% CI: 0.37-0.74). Cases with higher maternal body mass index (BMI) were 1.02 times more likely for macrosomia (95% CI: 1.01-1.04), and male infant sex (OR=1.78, 95% CI: 1.21-2.60). LBW was related to multiparity (OR=0.59, 95% CI: 0.42-0.82), multiple pregnancy (OR=17.35, 95% CI: 9.73-30.94), and preeclampsia (OR=3.36, 95% CI: 2.15-5.24).

**Conclusion:**

Maternal age, SES, preeclampsia, multiple pregnancy, ART, higher maternal BMI, parity, and male infant sex were determined to be predictive variables for birth weight and gestational age after taking into consideration their association by using a joint multilevel multiple logistic regression model

## Introduction

World Health Organization definitions on gestational age state that a baby is preterm if born before 37 weeks of gestation, full term if born between 37 through 42 weeks, and late or postterm if born after 42 weeks from the first day of the women’s last menstrual period (1, 2). Preterm birth is one of the leading risk factors of infant mortality in which children under 5 years of age are at a higher risk of death (3). Preterm birth is followed by permanent adverse consequences such as increased risk of impaired learning, cerebral palsy, and visual disorders. Chronic disease in adulthood can be an outcome of a preterm birth (4, 5). 

Preterm birth may result from risk factors that include multiple pregnancy, infection, advanced maternal age, short interval between pregnancies, low maternal body mass index (BMI), poor maternal nutrition, the use of assisted reproductive technology (ART), maternal psychological health, and lifestyle (6). The prevalence of preterm and postterm births range from 5 to 18% across developed and developing countries (5, 7). 

Currently, abnormal gestational age is more common due to the increased rate of multiple births, use of ART, and higher numbers of obstetric interventions (5, 8). In addition, postterm births can cause risks for both the mother and the infant such as fetal and neonatal mortality and morbidity, increased maternal morbidity, fetal macrosomia, placental insufficiency, meconium aspiration syndrome, and meconium aspiration (1). Potential reasons for postterm births include nulliparity, maternal age, race, and previous pregnancies with postterm deliveries or anencephaly (9).

Birth weight, or the first weight of a newborn baby, can be categorized as normal (=2.5 to < 4.0 kg), low (<2.5 kg), and macrosomia (=4.0 kg) (10). In addition to gestational age, abnormalities in birth weight have negative outcomes for the infant such as higher risk of infant mortality and inappropriate growth velocity for age. Similar to preterm, low birth weight (LBW) is associated with tobacco smoke, drug and alcohol consumption, anemia, bacterial vaginosis, short birth intervals, low BMI, teenage pregnancy, stress, and certain occupational factors (11-13). 

Macrosomia is associated with an increased risk of cesarean delivery, prolonged labor, perinatal trauma, maternal diabetes and obesity, excessive weight gain, male infant sex, prolonged gestation, high maternal age, multiparity, and postpartum hemorrhage. Adverse outcomes of macrosomia for the infant include dystocia, birth injury, or death. In addition, these children will be at risk for diabetes and obesity later in life (14, 15). LBW is more prevalent than macrosomia; however, the prevalence of macrosomia in developed countries is between 5% and 20%. In developing countries, macrosomia ranges from 0.5 to 15% (10, 16). The increasing trend of macrosomia in the last two decades may be due to the increasing rate of diabetes and obesity among reproductive age women (14, 16). Birth weight is strongly associated with gestational age so that prevention of preterm births reduces the risk for LBW (16, 17). 

Cluster data are widely recorded in medical and clinical areas where the cases are nested in clusters. For instance, students may be clustered in schools. In contrast to the traditional statistical approaches, multilevel models take the correlation among subjects in the same cluster into account (18, 19). Recording and analyzing two or more response variables in the same dataset is widely applied. Jointly analyzing several response variables prevents type I error inflation and increases the statistical power (20). 

The detrimental outcomes after abnormalities in birth weight and gestational age are well discussed in the literature. However, regarding the association between these two variables, it is very important to predict the classes of birth weight and gestational age using their potential risk factors. Hence, the current study aims to model birth weight and gestational age jointly for the data from maternity clinic centers in Tehran province by applying a joint multilevel multiple logistic regression model.

## Materials and Methods

### Participants and study design

We conducted this cross-sectional study on 4415 fertile women who referred to maternity clinic centers in Tehran Province, Iran, from July 6-21, 2015. These centers are supervised by the following universities located in Tehran, Iran: Tehran University of Medical Sciences, Shahid Beheshti University of Medical Sciences, Iran University of Medical Science, and Islamic Azad University School of Medicine.

### Ethical consideration

The Ethical Committee of Royan Institute approved our study. Patients received a clear explanation of the study goals as well as assurances for data confidentiality. The participants were assured that their choice to participate in the study would not affect their treatment procedures. Voluntary completion of the questionnaire was considered to be written informed consent.

### Questionnaires and variables

The instrument used in this survey was based on a checklist that consisted of the mothers’ demographics and information about midwifery and the infant. We completed the checklist by interviewing the mother; medical files in the delivery room were checked by a midwife and well-trained nurse. The checklist contained information about the maternal and paternal age (years), socioeconomic status (SES), mother’s BMI (kg/m^2^), baby’s head circumference (cm), parity (1 and ≥ 2), education of mother (undergraduate and graduate), mother’s occupation (housewife or employed), type of pregnancy (wanted, unwanted), history of abortion (yes or no), history of stillbirth (yes or no), preeclampsia (yes or no), multiple pregnancy (yes or no), and ART (yes or no).

### Outcome measures

i. Birth weight (g): LBW (<2500 g), normal birth weight (2500-4000 g) and macrosomia (≥4000 g) and ii. Gestational age (weeks) at birth: preterm birth (<37 weeks), term birth (37-42 weeks) and postterm birth (≥42 weeks of gestation). 

### Statistical analysis

We performed a joint multilevel multiple logistic regression model (18, 20). “Joint” refers to simultaneous modeling of two associated response variables. “Multilevel” refers to the two levels of cases are nested in the hospitals. “Multiple” refers to several predictors in the model. The pregnant women were considered to be the first level whereas hospitals comprised the second level. The correlation between the response variables is induced through random intercepts in each sub-model. The random intercepts are assumed to follow bivariate Bridge distribution with correlation parameters. In contrast to the normal, Bridge distribution provides researchers with the same odds ratio (OR) interpretation both within and between clusters (21). In order to link the systematic component with the response variables, a logit link function is considered. The model is specified as follows:

$$㏒\left(\frac{\mathrm{pr}\left(y_{\mathrm{1ij}},=,\mathrm{preterm or postterm}\right)}{\mathrm{pr}\left(y_{\mathrm{2ij}},=,\mathrm{term}\right)}\right)=u_{\mathrm{1i}}+\omega_{c}-x_{\mathrm{I'J}}\alpha$$

$$㏒\left(\frac{\mathrm{pr}\left(y_{\mathrm{2ij}},=,\mathrm{LBW or Macrosomia}\right)}{\mathrm{pr}\left(y_{\mathrm{2ij}},=,\mathrm{Normal}\right)}\right)=u_{\mathrm{2i}}+\theta_{c}-z_{\mathrm{I'J}}\beta$$

In the model, y1iJ and y2iJ are the gestational age and birth weight for subject j at hospital I, respectively. Both x and z are predictor variables of the response variables. â and á are the estimated coefficient vectors that correspond to the predictors. The terms u1i and u2i are the random intercepts (hospital specific effects). ù and è are the thresholds of each response variable category (c). The reference level for gestational age was “term” as well as “normal weight” for birth weight. According to the logit link function, eâ (eá) indicates the OR of LBW (preterm)/macrosomia (postterm) to normal weight (term) for X=x+1 compared to X=x. A 95% confidence interval (CI) that contained 1 indicated a P>0.05 and a non-significant effect size. Simple univariate multilevel nominal logistic regression models were separately applied for predictors. Those variables with P<0.20 were used in the joint multilevel multiple logistic regression model.

The data analysis was carried out recruiting the PROC NLMIXED in SAS software version 9.2 (SAS Institute, Inc.). A P<0.05 was considered significant. Two-sided tests were run for the statistical hypothesis.

## Results

We observed the following prevalence rates for preterm (5.5%), term (94%), and postterm (0.5%). LBW had a prevalence rate of 4.8%. The prevalence rate for normal weight was 92.4 and 2.8% for macrosomia. The independent chi-square test exposed a strong association between gestational age and weight at birth (Pearson chi-square=940.308, df=4, P<0.001).

The distribution of cases’ characteristics in three groups of gestational age and weight at birth are illustrated (Tables1, 2). Patients had a mean ± SD age of 29.18 ± 5.35 years. 

The joint multilevel multiple logistic regression model analysis adjusted the association between gestational age and birth weight as well as the interaction among several predictors (Table 3). Based on several simple univariate multilevel logistic regression models, we entered mother’s age and BMI, SES, mother’s education, preeclampsia, ART, multiple pregnancy, parity, history of stillbirth, and infant sex into the joint model. The joint multiple multilevel logistic regression model was fitted to the data (Table 3). A comparison of preterm to term in the joint model showed that mother’s age, preeclampsia, multiple pregnancy, and ART were significant predictors. Postterm to term comparison showed that mother’s age and SES were significant predictors. The OR of preterm to term among older mothers was strongly more likely than younger mothers (OR=1.04, 95% CI: 1.02-1.07) while postterm was not affected by mother’s age (OR=0.92, 95% CI: 0.85-1.01). Mothers with a better SES were less prone to have postterm births compared to term births (OR=0.53, 95% CI: 0.37-0.74). Mothers with preeclampsia were 4.14 (95% CI: 2.71-6.31) times more likely to experience preterm than term births. Patients with multiple pregnancy were 18.04 (95% CI: 9.75-33.38) times more prone to undergo preterm delivery compared to term delivery. Mothers in the ART group were 2.47 (95% CI: 1.64-33.73) times more prone to experience a preterm rather than term delivery.

We assessed infant weight at birth, mother’s age, mother’s BMI, mother’s education, parity, history of stillbirth, multiple pregnancy, preeclampsia, and infant sex as the candidate affective predictors by the univariate models. According to 95% CI, parity, multiple pregnancy, and preeclampsia had a statistical association with LBW. Macrosomia showed a significant association with mother’s BMI and infant sex. Mothers with more than two pregnancies were less likely to deliver a child with LBW (OR=0.59, 95% CI: 0.42-0.82). The patients with multiple pregnancy were 17.35 (95% CI: 9.73-30.94) times more prone for LBW than normal weight. Children from mothers with preeclampsia were 3.36 (95% CI: 2.15- 5.24) times more likely to experience LBW compared to normal mothers. The OR of macrosomia to normal weight for a mother with a higher BMI was 1.02 (95% CI: 1.01-1.04). Macrosomia was more common among male infants compared to females (OR= 1.78, 95% CI: 1.21-2.60).

**Table 1:** Distribution of patients’ characteristics in the preterm, term and postterm groups


Variable		Preterm n=244	Term n=4149	Postterm n=22	P value

Mother’s age (Y)		30.51 ± 5.95	29.11 ± 5.29	26.86 ± 6.36	<0.001
Father’s age (Y)		34.64 ± 6.29	33.43 ± 5.73	33.05 ± 7.11	0.006
SES		0.16 ± 2.11	0.03 ± 2.02	-1.93 ± 1.57	<0.001
Mother’s BMI (kg/m^2^)		25.01 ± 4.12	24.99 ± 5.60	24.10 ± 5.31	0.752
Baby’s head circumference (cm)		32.78 ± 2.81	34.98 ± 4.93	35.36 ± 1.18	<0.001
Parity					0.816
	1	122 (5.6)	2026 (93.8)	12 (0.6)	
	≥2	122 (5.4)	2123 (94.1)	10 (0.4)	
Mother’s education					0.007
	Undergraduate	149 (5)	2800 (94.3)	20 (0.7)	
	Graduate	95 (6.6)	1349 (93.3)	2 (0.1)	
Father’s education					0.253
	Undergraduate	156 (5.2)	2827 (94.2)	17 (0.6)	
	Graduate	88 (6.2)	1322 (93.4)	5 (0.4)	
Mother’s occupation					0.324
	Housewife	209 (5.4)	3645 (94.1)	21 (0.5)	
	Employed	35 (6.5)	504 (93.3)	1 (0.2)	
Type of pregnancy					0.882
	Wanted	194 (5.4)	3351 (94)	18 (0.5)	
	Unwanted	50 (5.9)	798 (93.7)	4 (0.5)	
History of abortion					0.251
	No	190 (5.3)	3353 (94.1)	20 (0.6)	
	Yes	54 (6.3)	796 (93.4)	2 (0.2)	
History of stillbirth					0.354
	No	237 (5.5)	4079 (94)	22 (0.5)	
	Yes	7 (9.1)	70 (90.9)	0 (0)	
Preeclampsia					<0.001
	No	198 (4.7)	3961 (94.8)	21 (0.5)	
	Yes	46 (19.6)	188 (80)	1 (0.4)	
ART					<0.001
	No	197 (4.8)	3867 (94.7)	19 (0.5)	
	Yes	47 (14.2)	282 (84.9)	3 (0.9)	
Infant sex					0.113
	Female	108 (5)	2054 (94.4)	14 (0.6)	
	Male	136 (6.1)	2095 (93.6)	8 (0.4)	
Multiple pregnancy					<0.001
	No	210 (4.8)	4121 (94.7)	22 (0.5)	
	Yes	34 (54.8)	28 (45.2)	0 (0)	
Birth weight					<0.001
	LBW	111 (52.1)	102 (47.9)	0 (0)	
	Normal	131 (3.2)	3928 (96.3)	19 (0.5)	
	Macrosomia	2 (1.6)	119 (96)	3 (2.4)	


Values are given as mean ± SD or number (%). SES; Socioeconomic status, BMI; Body mass index, ART; Assisted reproductive technology, and LBW; Low birth weight.

**Table 2:** Distribution of patients’ characteristics in the LBW, normal, and macrosomia groups


Variable		LBW n=213	Normal n=4078	Macrosomia n=124	P value

Mother’s age (Y)		29.40 ± 5.61	29.15 ± 5.35	29.90 ± 4.73	0.247
Father’s age (Y)		33.75 ± 6.41	33.44 ± 5.74	34.96 ± 5.61	0.013
SES		0.13 ± 2.01	0.02 ± 2.03	0.12 ± 1.93	0.625
Mother’s BMI (kg/m^2^)		24.98 ± 12.71	24.94 ± 4.92	26.49 ± 3.81	0.009
Baby’s head circumference (cm)		32.01 ± 2.39	34.96 ± 4.96	36.59 ± 1.43	<0.001
Parity					
	1	123 (5.7)	1983 (91.8)	54 (2.5)	0.016
	≥2	90 (4)	2095 (92.9)	70 (3.1)	
Mother’s education					0.176
	Undergraduate	13 (4.4)	2756 (92.8)	82 (2.8)	
	Graduate	82 (5.7)	1322 (91.4)	42 (2.9)	
Father’s education					0.155
	Undergraduate	142 (4.7)	2764 (92.1)	94 (3.1)	
	Graduate	71 (5)	1314 (92.9)	30 (2.1)	
Mother’s occupation					0.487
	Housewife	182 (4.7)	3582 (92.4)	111 (2.9)	
	Employed	31 (5.7)	496 (91.9)	13 (2.4)	
Type of pregnancy					0.449
	Wanted	179 (5)	3284 (92.2)	100 (2.8)	
	Unwanted	34 (4)	794 (93.2)	24 (2.8)	
History of abortion					0.957
	No	173 (4.9)	3238 (92.3)	101 (2.8)	
	Yes	40 (4.7)	789 (92.6)	23 (2.7)	
History of stillbirth					0.061
	No	207 (4.8)	4012 (92.5)	119 (2.7)	
	Yes	6 (7.8)	66 (85.7)	5 (6.5)	
Preeclampsia					<0.001
	No	178 (4.3)	3887 (93)	115 (2.8)	
	Yes	35 (14.9)	191 (81.3)	9 (3.8)	
ART					0.281
	No	191 (4.7)	3777 (92.5)	115 (2.8)	
	Yes	22 (6.6)	301 (90.7)	9 (2.7)	
Infant sex					0.009
	Female	112 (5.1)	2019 (92.8)	45 (2.1)	
	Male	101 (4.5)	2059 (92)	79 (3.5)	
Multiple pregnancy					<0.001
	No	185 (4.2)	4045 (92.9)	123 (2.8)	
	Yes	28 (45.2)	33 (53.2)	1 (1.6)	


Values are given as mean ± SD or number (%). SES; Socioeconomic status, BMI; Body mass index, ART; Assisted reproductive technology, and LBW; Low birth weight.

**Table 3:** The results of joint multilevel multiple logistic regression model determining gestational age and birth weight categories


Predictor		Preterm to termOR (95% CI)	Postterm to termOR (95% CI)

Mother’s age (Y)		1.04 (1.02-1.07)	0.92 (0.85-1.01)
SES		0.97 (0.89-1.07)	0.53 (0.37-0.74)
Mother’s education		1.29 (0.90-1.86)	0.82 (0.17-3.91)
Preeclampsia			
	Yes	4.14 (2.71-6.31)	109 (0.14-8.39)
	No	Reference category	
ART			
	Yes	2.47 (1.64-33.73)	109 (0.14-8.39)
	No	Reference category	
Multiple pregnancy			
	Yes	18.04 (9.75-33.38)	0.61 (0.001-4.68)
	No	Reference category	
		**LBW to normal OR (95% CI)**	**Macrosomia to normal OR (95% CI)**
Mother’s age (Y)		1.01 (0.98-1.04)	1.01 (0.98-1.05)
Mother’s BMI (kg/m^2^)		1.01 (0.97-1.03)	1.02 (1.01-1.04)
Mother’s education			1.10 (0.72-1.69)
	Graduate	1.15 (0.82-1.61)	
	Underggraduate	Reference category	
History of stillbirth			
	Yes	2.17 (0.89-5.28)	2.47 (0.94-6.52)
	No	Reference category	
Multiple pregnancy			
	Yes	17.35 (9.73-30.94)	0.72 (0.08-6.84)
	No	Reference category	
Preeclampsia			
	Yes	3.36 (2.15-5.24)	1.4 (0.68-2.87)
	No	Reference category	
Infant sex			
	Male	0.87 (0.65-1.17)	1.78 (1.21-2.60)
	Female	Reference category	


SES; Socioeconomic status, BMI; Body mass index, ART; Assisted reproductive technology, OR; Odds ratio, and CI; Confidence interval.

## Discussion

Numerous studies reported a direct, positive correlation between weight at birth and gestational age (1, 13, 22, 23). We conducted the current study to determine the risk factors for the adverse categories of these two pregnancy outcomes regarding their association where the OR of preterm or postterm to term as well as LBW or macrosomia to normal birth weight have been demonstrated. Our study has shown that postterm birth did not have any association with mother’s age. However, older mothers are more prone to have preterm deliveries. This may be due to certain conditions at older ages such as elevated blood pressure. 

A systematic review Flenady et al. (24) has sought to determine whether older maternal age could be a risk factor for preterm birth. They reported that most studies indicated that preterm birth was significantly affected by older maternal age. The current study demonstrated that better SES reduced the chance of preterm or postterm delivery. This might be due to the fact that families with relatively better SES have greater access to facilities. Whitehead assessed the relationship between SES and preterm delivery and contractions. SES appeared to be associated with the basic factors of spontaneous preterm delivery, but not directly with preterm delivery (25). 

Kistka et al. (26) assessed the risk for postterm delivery. They showed that low SES scores had an association with increased risk for recurrent postterm birth. Joseph et al. (27) assessed the relationship between spontaneous preterm and socioeconomic position; they observed significantly higher preterm births among low income families.

We showed that the presence of preeclampsia increased the odds for preterm and LBW. However, our study showed that the odds for postterm delivery and macrosomia were higher among those with preeclampsia. These ratios were not significant. Davies et al. (28) investigated the association between preeclampsia and preterm in a large population of primiparous women. Their study confirmed that preeclampsia contributed to preterm delivery and reduction in preterm rates could be achieved by controlling preeclampsia. The same results were reported by Goldenberg et al. (23). In contrast to postterm births, preterm births had a significant association with the use of ART. 

Dunietz et al. (29) assessed the association of preterm delivery and ART among primiparous women. They demonstrated that the use of ART increased the risk for preterm birth, even in the cases with male factor infertility. Our results revealed that multiple pregnancy significantly predicted preterm birth and LBW, but did not affect postterm and macrosomia. It has been shown that the majority of twins are born preterm. The relationship between preterm and multiple pregnancy was well discussed by Liem et al. (30). The leading cause of preterm births was over-distension of the uterus (31). 

Based on the results from our study, macrosomia was affected by mother’s BMI. Rockhill et al. (32) reported the same findings when they assessed the effects of pre- pregnancy BMI on fetal macrosomia. Jolly et al. (33) studied affected variables of macrosomia as well as its clinical outcomes using a large data on pregnancies. They demonstrated a higher rate of macrosomia among mothers with higher BMI. The current study also showed that male infant sex was more common in macrosomia. This result was consistent with the study from Ju et al. (34) who assessed fetal macrosomia and pregnancy outcomes. Although the current study had less numbers of birth results at more normal weight compared to macrosomia, this finding was not significant. However, increased numbers of births were associated with decreased odds for LBW. The same result was found by Nazari et al. They compared maternal characteristics in LBW and normal birth weight infants. They found that primiparous mothers were more at risk for LBW infants in comparison with multiparous mothers (12).

The strength of this study was the association between two ordinal outcomes of pregnancy and determining their potential risk factors by using an advanced statistical joint modeling approach. Ignoring the strong association between the two response variables, weight at birth and gestational age, reduced the statistical power to find their significant risk factors. In contrast to univariate models and traditional approaches, jointly modeling several response variables increases the statistical power of data analysis. In a study by Santos et al. (35), multivariate and univariate GARCH models were fitted to forecast portfolio value-at-risk. They determined that more valid results were provided by the multivariate approaches.

Kassahun et al. used a joint model for hierarchical continuous and zero-inflated overdispersed count data to assess the diarrhoeal disease burden. To do so, the combined infant body weight and number of days of diarrhoeal illness using a longitudinal design (36). Moreover, a multilevel structure of a dataset has been shown to cause some variances in which multilevel modeling approaches must be applied (37). 

These types of data are widely used in medical and clinical areas; ignoring its’ natural variance causes misleading estimations (19). Nkansah-Amankra et al. (38) have evaluated the effects of maternal stress on LBW and preterm birth outcomes using multilevel logistic analysis. The multilevel analysis simultaneously modeled individual and neighborhood contexts to determine the odds of LBW and preterm delivery. In a similar study, Ota et al. (39) assessed the risk factors of preeclampsia and its adverse outcomes in low- and middle-income countries. They used multilevel regression models to determine the associations between preeclampsia and its risk factors.

## Conclusion

The study results showed an association between preterm and postterm births to maternal age and SES. In contrast to postterm births, preeclampsia, multiple pregnancy, and ART affected preterm births. Macrosomia was caused by higher maternal BMI. Macrosomia was more common among male infants. We observed an association between LBW and parity, preeclampsia, and multiple pregnancy. We have determined that the joint multilevel multiple logistic regression model is a proper statistical tool for these types of data. 
